# Genetic and Epigenetic Alterations in Autism Spectrum Disorder

**DOI:** 10.1055/s-0041-1735540

**Published:** 2021-09-15

**Authors:** Bugsem Oztenekecioglu, Merdiye Mavis, Meryem Osum, Rasime Kalkan

**Affiliations:** 1 Department of Molecular Biology and Genetics, Faculty of Arts and Sciences, Near East University, Nicosia, Cyprus; 2 Department of Medical Genetics, Faculty of Medicine, Near East University, Nicosia, Cyprus

**Keywords:** genetic, epigenetic, autism spectrum disorder

## Abstract

It is extremely important to understand the causes of autism spectrum disorder (ASD) which is a neurodevelopmental disease. Treatment and lifelong support of autism are also important to improve the patient's life quality. In this article, several findings were explained to understand the possible causes of ASD. We draw, outline, and describe ASD and its relation with the epigenetic mechanisms. Here, we discuss, several different factors leading to ASD such as environmental, epigenetic, and genetic factors.

## Introduction

Autism spectrum disorder (ASD) is a neurodevelopmental disease that can be either a congenital or early disease that occurs due to nervous system abnormalities. Since autism arises from nervous system abnormalities, it affects the structure or functioning of the brain. Accordingly, due to the abnormal electrical movements within the brain, neurological problems such as seizures, involuntary movements, and loss of consciousness may occur. Individuals who suffered from ASD face significant problems in social life, school, or at work. Besides, individuals diagnosed with ASD have features such as overwhelming memory, the ability to play music and sing. In ASD, the word “spectrum” stands for a wide range of symptoms and to what extent these symptoms progress and the degree of symptoms develop. 1 An infant or an adult with ASD can have varying social behavior and coping skills issues. For example, they avoid eye contact and lack a particular facial expression. They do not react to being named either they do not seem to hear individuals speaking to them from time to time. They do not talk, speak late, or lose the ability to say previously acquired words or sentences. They are resisting to hug and shake hands, prefer to play alone, retreat to their world, not understanding how to use words or sentences despite repeating word for word, repeating the same word continuously. With their behavioral habits, an infant or adult with ASD can have multiple concerns. These are repetitive movements such as swinging back and forth, turning around or clapping hands, coordination problems, indifference to pain or temperature despite being unusually sensitive to light, sound, or contact, abnormally focused or stuck on an object or activity, refusing certain types of food altogether or eating only certain foods. A child or adult with ASD may have different issues with repeated forms of behavior such as serotypes and repeated motor movements, insistence on sameness, strict obedience to schedule, limited and extreme interest, and sensory issues of less or more arousal. 2

## Etiology of Autism Spectrum Disorder

### Environmental Factors

Environmental factors can cause ASD such as viral infections or drugs are taken during pregnancy since the placenta is an imperfect barrier that limits what goes into the fetus, and therefore, it partially protects the fetus. Materials or chemicals can cross the placenta; small nonpolar molecules that do not easily dissolve in water can cross the placenta easily, while large polar molecules can cross poorly. 3 Those chemicals increase DNA methylation, providing evidence of possible epigenetic changes involved in ASD. Depending on the country, drugs have different kinds of testing procedures, and various companies have tested and categorized drugs in regard to how safe they are to be used during pregnancy. 4 There is a threshold and lower than the threshold effect, usually it is not expected to have an anomaly for the fetus. 5 Also, gestation age is important to consider in case of exposure to teratogens. The fetus gets affected by teratogens during organ development. For that reason, some sources of teratogens may compromise the normal development of an organ. There is also growing evidence that air pollution might be one of the factors causing autism. Every chemical substance may be teratogenic as well as it will depend on how much the mother consumes it. 6 The lifestyle of a pregnant woman is important. During pregnancy, if she is smoking, using alcohol, this might cause several problems in the fetus. Adopting a healthy diet is also important for the health of the fetus. The effects of those can occur during postnatal or prenatal life, which means that if the mother has been exposed to teratogens during pregnancy that will lead to anomalies in brain development or several health problems of the fetus. 7 Parental age at conception may also be associated with an increased risk of autism. Consequently, serious problems such as prematurity or oxygen deprivation may be faced. 8

### Genetic Factors

Autistic spectrum disorder is a complex disorder and multiple factors such as genetic factors, epigenetic modifications, and environmental factors are involved in the development of the autism disorder. Therefore, the inheritance pattern is not fully known. 9 10 ASD can be caused by a microdeletion of the 16p11.2 chromosome. Several genes have been identified associated with autism, including the *PTCHD1* , 9 11 *HOX* , *CHD2* , *CHD8* , *FOXP2* , *SHANK3* , and *OXTR* genes ( Table 1 ). 12

**Table 1:** Summary of autism disorder-related genes

Genes	Causes
*PTCHD1*	Mental development
*HOX*	Significant for brain development
*CHD8*	Deletion or inversion causes brain abnormalities
*FOXP2*	Developmental language and speech deficiencies
*SHANK3*	Neurotransmitter receptors and nerve cell communication
*OXTR*	Impact on face memory

The deletion and/or inversion of the *CDH8* gene, which has located on the long arm of chromosome 16, can cause autism disorder. 13 *SHANK2* gene localized on the long arm of chromosome 22 and can cause autism disorder. Alterations of the *SHANK2* gene causes structural organization problems of neurotransmitter receptors and nerve cell communication. 14 *FOXP2* is associated with developmental language and speech deficiencies. 15 Mutations on homeotic genes caused brain abnormalities. 16 *OXTR* , known as oxytocin receptor, is a protein and acts as a receptor for the hormone and neurotransmitter oxytocin. Mutations of the *OXTR* gene had an important impact on face memory. Low oxytocin and estrogen activation do not inhibit the methylation of the oxytocin receptor and silencing contributes to the enhanced amygdala and antisocial activity arousal intakes regulated by the androgen receptor. 17

SHANK proteins connect neurotransmitter receptors, ion channels, and other membrane proteins to cytoskeleton actin and signaling proteins. They play a key role during synapse formation and dendritic spine maturation, 18 and variations of the *SHANK2* gene have been identified in individuals with ASD. 19 Several studies identified various candidate genes which they have a possible causal role in ASD pathogenesis such as voltage-gated ion channels, scaffold proteins, cell adhesion molecules, and synaptic architecture. 20 Mutations in *CACNA1C* , *CACNA1D* , *CACNA1E* , *CACNA1F* , *CACNA1H* , *SCN1A* , *SCN2A* , *SCN3A* , *SCN7A* , *SNC8A* , *KCNMA1* , *KCND2* , *KCNJ10* , *KCNQ3* , and *KCNQ5* have been described in ASD. 20

### Epigenetic Factors

In addition to environmental factors, findings are suggesting another significant factor causing autism may be genetics or epigenetic. Epigenetics are the heritable or acquired changes in gene expression without modifications in the sequence of DNA. It has a different level of gene expression. Epigenetic alterations play an important role and can change gene expression levels without changing genomic DNA. However, they can change the gene expression level; there will be increased or decreased gene expression. Four different mechanisms of epigenetic modifications are histone modifications, DNA methylation, RNA interference, and RNA modifications. They play essential roles at the level of gene expression and transcription. Although the chromatin shifts that have just been addressed do not affect the DNA code, they can be carried on to the subsequent generations of the cell. Epigenetic inheritance is also the inheritance of characteristics inherited through pathways not specifically affecting the nucleotide sequence. Epigenetic means individuals have alterations with inheritable parents. They do not show Mendelian inheritance pattern. Therefore, it includes chromatin structure instead is related to different packaging levels. Epigenetic pathways involving DNA methylation, posttranslational modifications to histone proteins, and transcriptional regulations that are central to neurodevelopmental processes in utero are likely to be affected by maternal habits such as obesity, hunger, smoking, and alcohol. 21

### Histone Modifications

Histone modifications can help for the segmentation of the genome into domains of different transcriptional potentials. Nucleosomal histones are subjected to a variety of various modifications affecting the particular residues of amino acids in the histone tails. Different chemical groups can be added; acetylation, monomethylation, dimethylation, or trimethylation of lysine and phosphorylation of serine into the histone tails and those modifications can attach histone tails covalently with the help of effective proteins called bromodomains. Depending on the nature of the group, they affect the level of DNA expression. Histone modification occurs through acetylation or methylation of the histone tail. Histone tail acetylation allows the nucleosome to dissolve, releasing the gene and altering the composition of the DNA. In comparison to acetylation, histone tail methylation tightens the nucleosomes together, inactivates the gene, and leaves DNA unapproachable for alteration. By adding the methyl group to cytosine, blocking the transcription of those genes, methylation may also occur. 22 Histone acetylome-wide association study analyzed samples from the patients with ASD and combined with the control brains that were exposed to H3K27 chromatin sequencing. 23 According to this acetylome study, cis regulatory elements were observed with a high rate in patients with ASD. There was a link between genes and ion channels. This was related to epilepsy and neuronal impulses. It was shown to be irregular during ASD. In addition, reduced acetylation in ASD can be caused by conditions, for example, chemokine signaling and histone deacetylase activity. They are corepressor that enhance the activities of transcriptional repressors. HDAC removes the acetyl group on the histone tail and changes chromatin configuration. Therefore, it contains a low level of acetyl group so that gene expression does not occur because they are highly packaged. 21

### DNA Methylation

DNA methylation, the addition of methyl marks on the fifth carbon of cytosine, affects the expression status of genes. DNA methylation is directly related to DNA expression and transcription level. Methylation plays a role during the differentiation, regulation of gene expression, 12 several disorders including cancer, imprinting disorders, Rett's syndrome, fragile X syndrome, tuberous sclerosis, or Angelman's syndrome. 24 *UBE3A* gene, located on chromosome 15q11–13, and mutations were observed in Angelman's syndrome. Also, studies showed that individuals who have three or too many copies of *UBE3A* have an increased risk for ASD. However, duplication on chromosome 15q11–13 leads to repetitive self-grooming behavior. Glutamate delivery was suffered because of three copies of the *UBE3A* gene. This neurotransmitter, which has been related to autism, is important for excitatory signaling. *UBE3A* is the most common gene for hereditary causes of autism. 25 Rett's syndrome is related to different abnormalities and neural development. *MECP2* gene, methylated cytosine-binding protein, plays a significant role in Rett's syndrome and is also a central contributor to neurological disorders, stimulates and suppresses transcription ( Table 2 ). 26

**Table 2:** Epigenetic alterations of autism spectrum disorder

Epigenetic factors	Causes
RNA methylation	Presumably deregulation of the regulatory transition between mRNA turnover and protein synthesis. Alterations in the methylation lead to decreased methylation capability and can also minimize RNA methylation.
IncRNAs	Differentially expressed IncRNAs are involved in the failure of neuronal connectivity and synaptic functions. IncRNAs overlap with the imprinted loci. IncRNAs (e.g., MSNP1AS) are associated with the risk of ASD IncRNA.
miRNAs	It controls genes involved in synaptic mechanisms, synaptic plasticity and memory, neuronal morphology and functions. Linked with a variety of cell signal pathways. For predictive or diagnosis of autism biomarkers, circulating serum and salivary miRNAs holds promise into the future.
Inhibition of HDAC1	HDAC1 valproic acid inhibition and GSK3B lithium upregulate the Wnt transcription inhibition and lead to macrocephalus.
Histone H3 phosphorylation	HMT LSD1, which prevents H3K4 demethylation for the stimulation of the androgen receptor gene, contributes to AR gene activation loss, raises the amygdala's elevated arousal consumption.
Epigenetic proteins	Decreased expression of DNA methyltransferase.

Recent studies showed that mutations of the epigenetic gene *ASH1L* were associated with ASD. Gao et al showed loss of ASH1L in the developing mouse brain caused multiple developmental defects, core autistic-like behaviors, and impaired cognitive memory. 25 ASH1L is a histone methyltransferase and mediates dimethylation of histone H3 lysine 36. Mutation studies showed that mutations of ASH1L were associated with ASD and/or intellectual disability. 27 28

Karayiorgou et al demonstrated differentially methylated sites in *NR3C1* , *MTHFR* , *DRD4* , *5-HTT* , *IGF2DMR* , *H19* , and *KCNQ1OT1* genes by using genome-wide DNA methylation in ASD patients. 29 Decreased level methylation of *PRRT1* and *TSPAN32* was observed in the cerebellum and temporal cortex of brains from 19 autism cases and 21 controls. 30 31 Stenz et al demonstrated hypomethylation of *ITGB2 (C3R)* , *SPI1* , *TNF-α* , *C1Q* , *IRF8m* , and *C3* genes in brain tissue samples from 13 autism cases and 12 controls. They demonstrated an adverse correlation between gene expression and DNA methylation within the individuals. 27 Hypomethylation of *RRT1* and *TSPAN32* genes were determined in the cerebellum and temporal cortex in the brains of ASD patients. 32 In addition to this studies, loss of PRRT1 was reported to increase the number of synapses and decrease synaptic strength that leads to synaptic dysfunction in ASD. 33

Several other studies have also examined the altered pattern of DNA methylation of other genes or evaluated the whole-genome methylation in ASD patients. All these studies demonstrated the importance of epigenetic studies during the identification of ASD pathophysiology. Altered methylation of neuronal regulation, synaptic signaling, and immune system–related genes can be useful for the identification of ASD-related biomarkers. The epigenetic, environmental, or genetic factors can cause ASD and advancement on diagnostic tools can be used for the identification of epigenetic alterations such as ASD-specific histone markers, regions between genes and noncoding RNAs in ASD. 21

## Conclusion

In conclusion, no definitive findings have yet been reached in the diagnosis of autism. However, there is more than one factor that causes autism. Genetic factors are among the studies that tried to identify the possible genetic background of autism. It has been observed that more than one gene was related to autism. However, although these findings are important, it should be considered that environmental and epigenetic factors play an essential role in many diseases such as autism. Candidate gene studies and alterations of epigenetic regulators highlight the importance of the interaction between genetic and environmental factors on the etiology of ASD. Gene and environment interactions are contributing to the increased prevalence of ASD. In that perspective, epigenetic modifications and environment interaction hold promise for possible biomarkers of autism. In line with these findings, many researchers have been researched to find out possible biomarkers of autism and increase the quality of life of affected individuals, reduce their symptoms, make them adapt to daily life, and increase and strengthen their attitudes in human relations.

Overall, alterations in the status of DNA methylation in patients with ASD have been discussed in this review article. Here, we described how DNA methylation patterns and genetic alterations are related to the pathophysiology of ASD. We suggest that our article shed light on future studies related to the possible biomarkers of autism.
